# CONTINUING CHALLENGES IN STATE EFFORTS TO IMPROVE DIRECT CARE WORKER WAGES

**DOI:** 10.1093/geroni/igad104.0773

**Published:** 2023-12-21

**Authors:** Denise Tyler, Melissa Hunter, Kristie Porter, Olga Khavjou, Marie Squillace, Judith Dey, Iara Oliveira

**Affiliations:** RTI International, Hancock, Maine, United States; RTI International, Durham, North Carolina, United States; RTI International, Durham, North Carolina, United States; RTI International, Raleigh, North Carolina, United States; Office of the Assistant Secretary for Planning and Evaluation, Washington, District of Columbia, United States; US Department of Health and Human Services, Washington, District of Columbia, United States; Office of Behavioral Health, Disability, and Aging Policy (BHDAP), Washington, District of Columbia, United States

## Abstract

Direct care workers (DCWs) receive low wages and some states have tried to improve their wages through policy changes. This mixed methods study examined the effect of state wage policies on changes in DCW wages. We also conducted 38 interviews with national experts and stakeholders across six states to determine the results of these policy changes and continued barriers to improving DCW wages. Results show some states that implemented policies to improve the wages of DCWs since 2009 reduced the gap between DCW wages and the wages of other entry-level workers, yet the gap was still substantial in many states. Experts and state stakeholders repeatedly cited consistent funding of wage policies through Medicaid reimbursement rate increases as important to effectively increasing and sustaining higher wages. Interviewees recommended that funding increases be continual, rather than requiring annual reauthorization and noted it was particularly challenging when funds were inadequate to support annual wage increases. Interviewees described Medicaid reimbursement rates as a barrier when rates did not keep pace with market trends. Interviewees also recommended professionalizing the field, increasing the respect afforded these workers, and developing ways to increase the pipeline of workers as additional ways to improve direct care work. Until there is meaningful policy change, states will continue to struggle with barriers to improved wages for DCWs which results in a disproportionate number of DCWs relying on public benefits and difficulties with recruitment and retention.

